# Supplementary figures and images for: Snf1 AMPK positively regulates ER-phagy via expression control of Atg39 autophagy receptor in yeast ER stress response
Source: PLoS Genet. 2020 Sep 28;16(9):e1009053. doi: 10.1371/journal.pgen.1009053 (PMC7544123; doi:10.1371/journal.pgen.1009053)

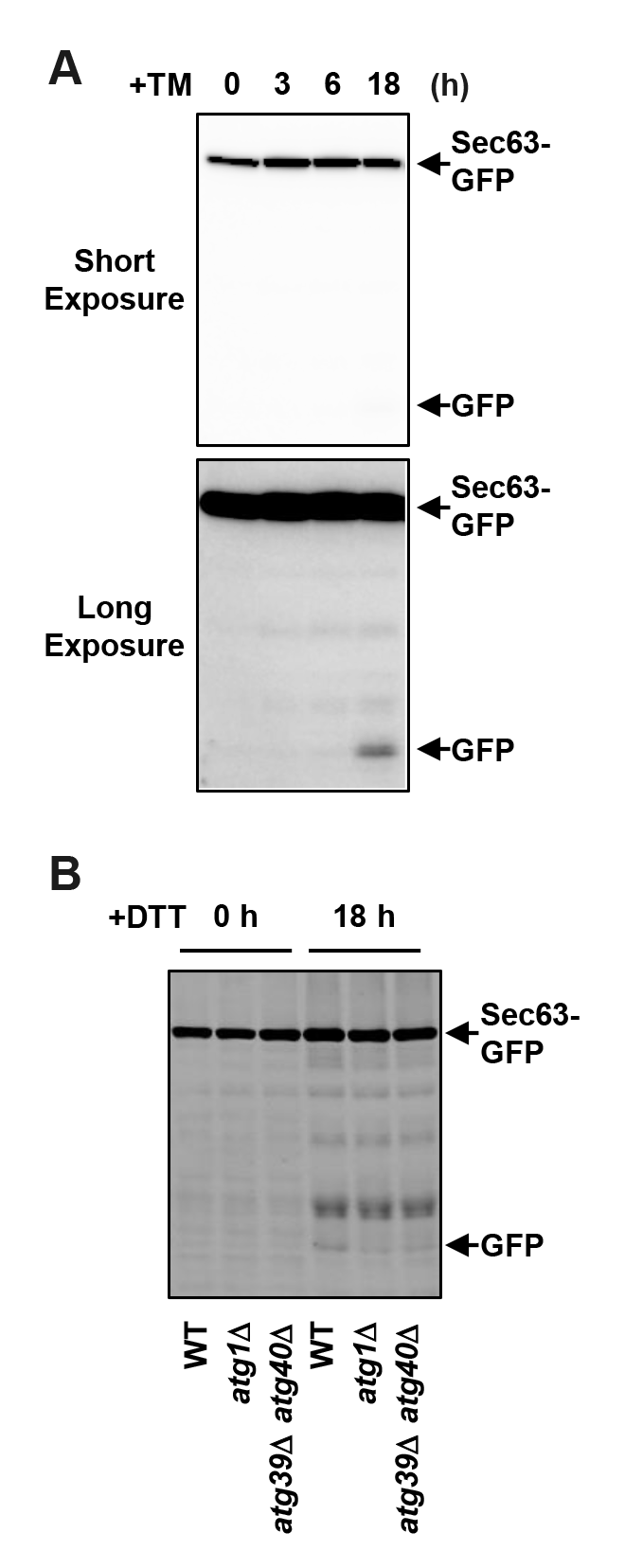

Supplement: S1 Fig — (A) Wild-type strains harboring GFP-tagged SEC63 were grown at 25 °C until exponential phase and treated with 3 μg/ml tunicamycin (TM) for the indicated time. Extracts prepared from each cell were immunoblotted with anti-GFP antibodies. (B) Wild-type (WT) and indicated mutant strains harboring GFP-tagged SEC63 were grown at 25 °C until exponential phase and treated with 6 mM dithiothreitol (DTT) for 18 hr. Extracts prepared from each cell were immunoblotted with anti-GFP antibodies. (TIF) [file pgen.1009053.s004.tif]

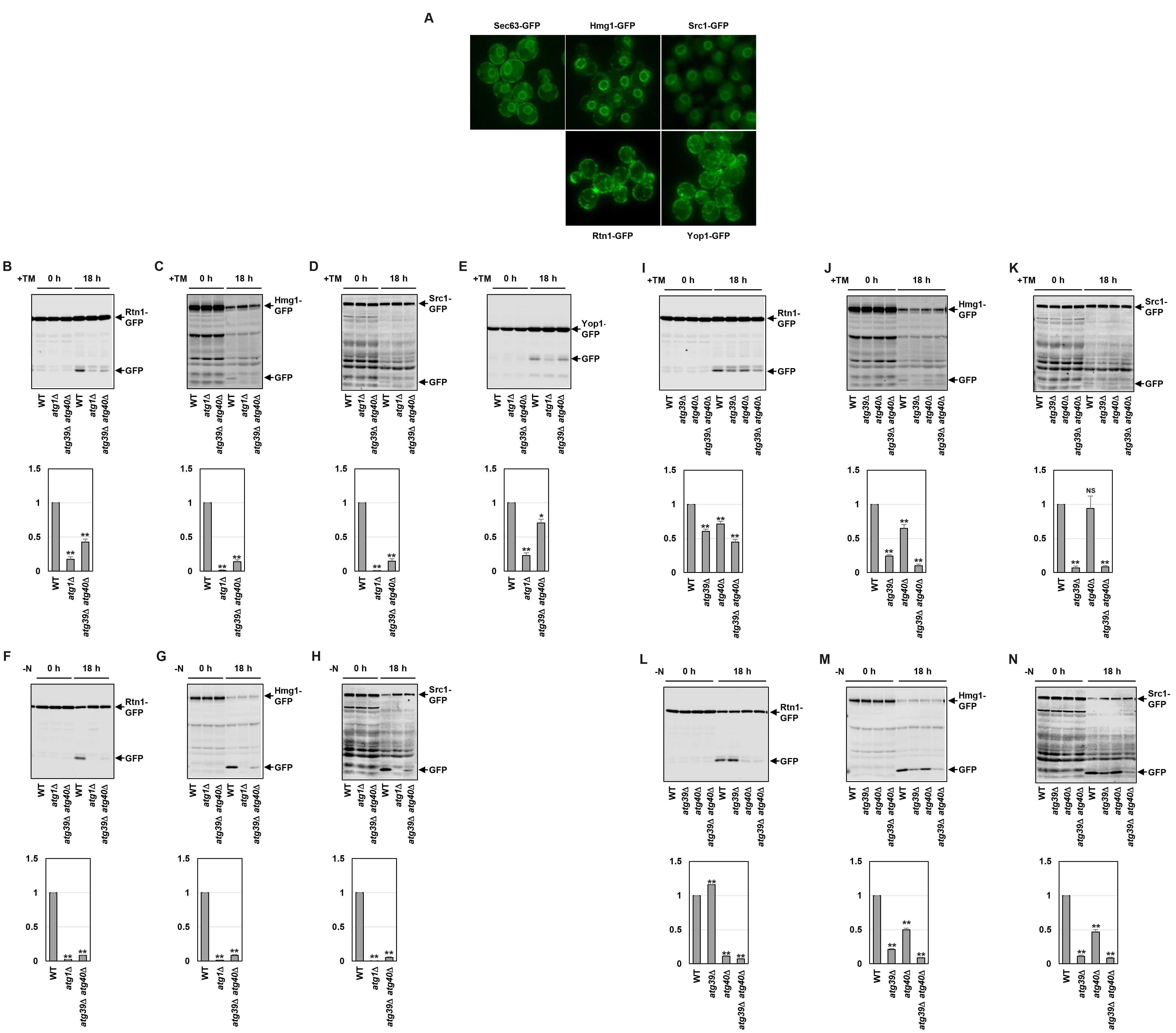

Supplement: S2 Fig — (A) Cellular localization of Sec63, Hmg1, Src1, Rtn1 and Yop1. Wild-type harboring GFP-tagged SEC63, HMG1, SRC1, RTN1 or YOP1 were grown at 25 °C until exponential phase and subjected to microscopy. (B-E) Degradation of Rtn1-GFP (B), Hmg1-GFP (C), Src1-GFP (D), and Yop1-GFP (E) after ER stress treatment. Wild-type (WT) and indicated mutant strains harboring GFP-tagged RTN1, HMG1, SRC1, or YOP1 were grown at 25 °C until exponential phase and treated with 3 μg/ml tunicamycin (TM) for 18 hr. Extracts prepared from each cell were immunoblotted with anti-GFP antibodies. The intensities of free GFP were measured and normalized to the intact GFP-tagged protein level. The values are plotted as the fold change from wild-type cells. The data show mean ± SEM (n > 3). *P < 0.05 and **P < 0.01 as determined by Student’s t-test. (F-H) Degradation of Rtn1-GFP (F), Hmg1-GFP (G), and Src1-GFP (H) after nitrogen starvation. Wild-type (WT) and indicated mutant strains harboring GFP-tagged RTN1, HMG1, or SRC1 were grown at 25 °C until exponential phase and then incubated under nitrogen-starved conditions for 18 hr. Extracts prepared from each cell were immunoblotted with anti-GFP antibodies. The intensities of free GFP were measured and normalized to the intact GFP-tagged protein level. The values are plotted as the fold change from wild-type cells. The data show mean ± SEM (n > 3). **P < 0.01 as determined by Student’s t-test. (I-K) Degradation of Rtn1-GFP (I), Hmg1-GFP (J), and Src1-GFP (K) after ER stress treatment. Wild-type (WT) and indicated mutant strains harboring GFP-tagged RTN1, HMG1, or SRC1 were grown at 25 °C until exponential phase and treated with 3 μg/ml tunicamycin (TM) for 18 hr. Extracts prepared from each cell were immunoblotted with anti-GFP antibodies. The intensities of free GFP were measured and normalized to the intact GFP-tagged protein level. The values are plotted as the fold change from wild-type cells. The data show mean ± SEM (n > 3). **P < 0.01 as determ [file pgen.1009053.s005.tif]

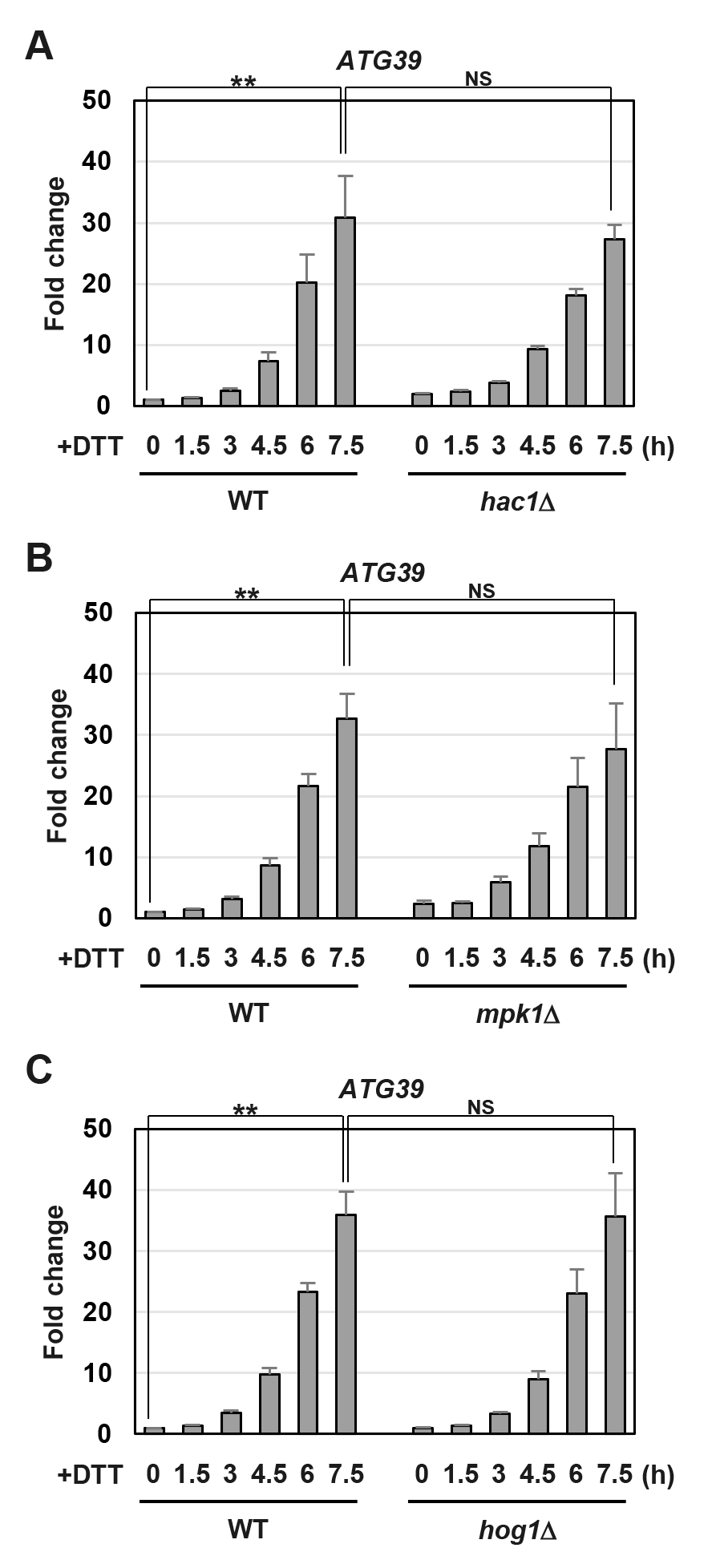

Supplement: S3 Fig — (A-C) Wild-type (WT) and indicated mutant strains were grown at 25 °C until exponential phase and treated with 6 mM dithiothreitol (DTT) for the indicated time. The ATG39 mRNA levels were quantified by qRT-PCR analysis, and relative mRNA levels were calculated using ACT1 mRNA. The values are plotted as the fold change from wild-type cells at the time of DTT addition. The data show mean ± SEM (n > 3). **P < 0.01 as determined by Student’s t-test. (TIF) [file pgen.1009053.s006.tif]

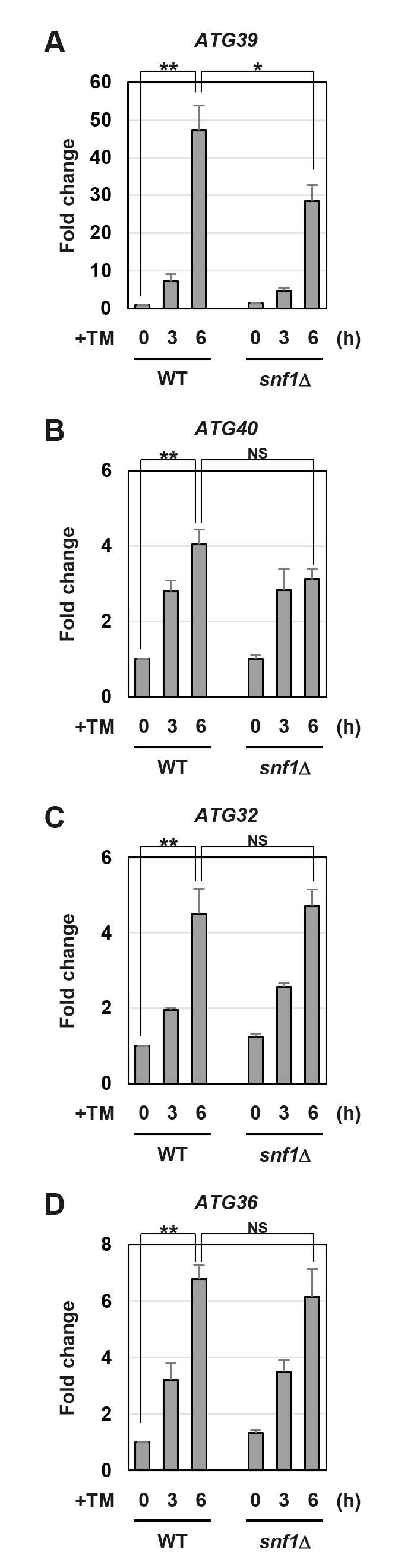

Supplement: S4 Fig — (A-D) Wild-type (WT) and snf1 mutant strains were grown at 25 °C until exponential phase and treated with 3 μg/ml tunicamycin (TM) for the indicated time. The mRNA levels were quantified by qRT-PCR analysis, and relative mRNA levels were calculated using ACT1 mRNA. The values are plotted as the fold change from wild-type cells at the time of TM addition. The data show mean ± SEM (n > 3). *P < 0.05 and **P < 0.01 as determined by Student’s t-test. NS, not statistically significant (P > 0.05). (TIF) [file pgen.1009053.s007.tif]

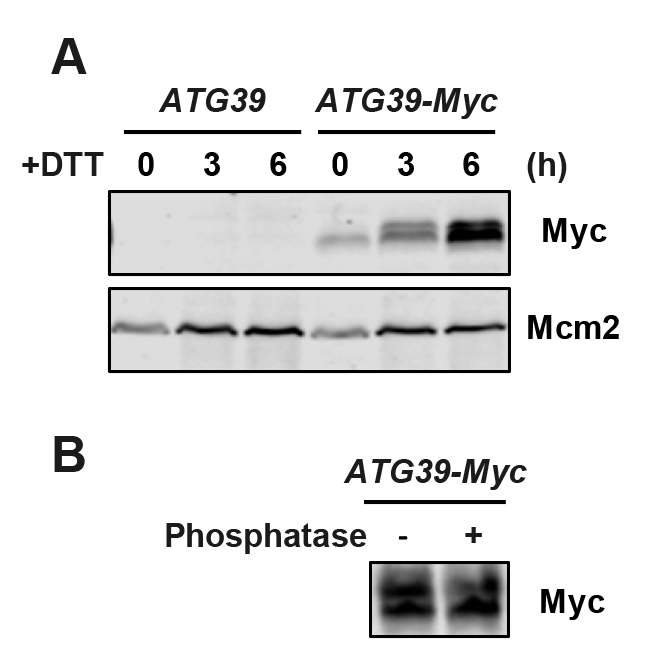

Supplement: S5 Fig — (A) The Atg39 protein level after ER stress treatment. Wild-type strains harboring non-tagged or Myc-tagged ATG39 were grown at 25 °C until exponential phase and treated with 6 mM dithiothreitol (DTT) for the indicated time. (B) Effects of the phosphatase treatment on Atg39. Wild-type strains harboring Myc-tagged ATG39 were grown at 25 °C until exponential phase and treated with 6 mM dithiothreitol for 6 hr. Extracts were treated with or without calf intestinal alkaline phosphatase and subjected to immunoblot with anti-Myc antibodies. (TIF) [file pgen.1009053.s008.tif]

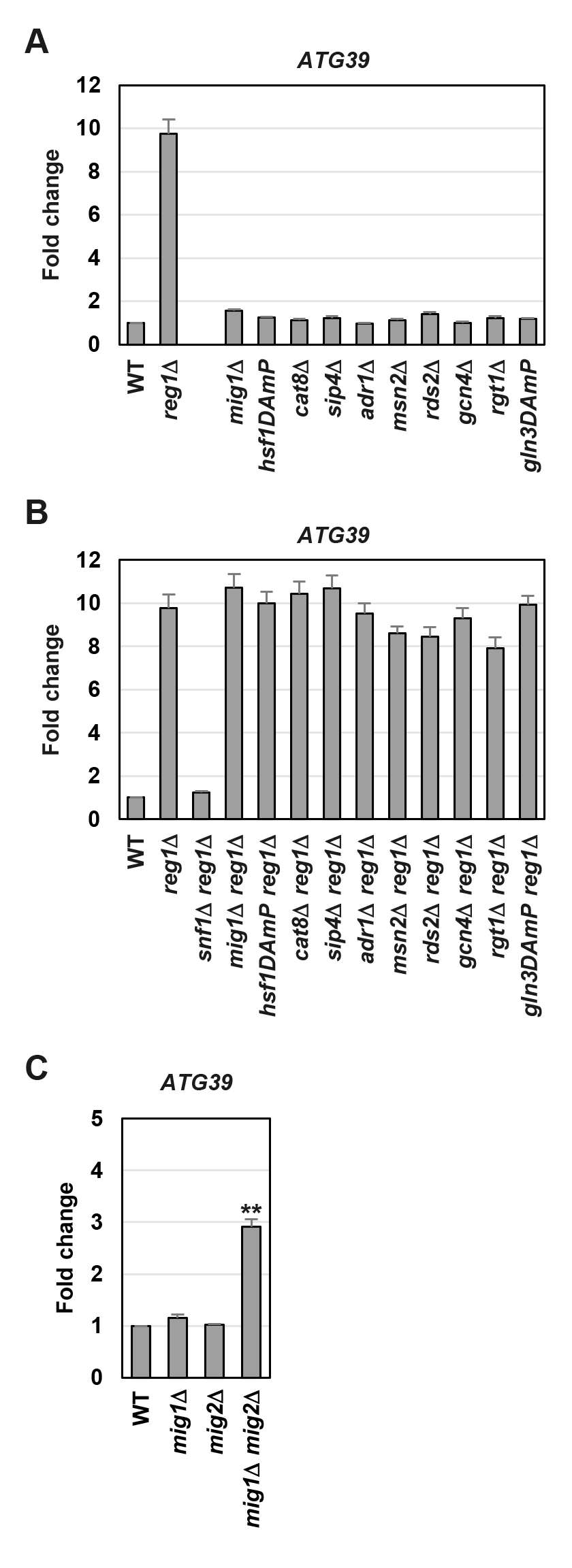

Supplement: S6 Fig — (A, B) The ATG39 mRNA levels in mutants of known Snf1 targets. Wild-type (WT) and indicated mutant strains were grown at 25 °C until exponential phase. The ATG39 mRNA levels were quantified by qRT-PCR analysis, and relative mRNA levels were calculated using ACT1 mRNA. The values are plotted as the fold change from wild-type cells. The data show mean ± SEM (n = 3). (C) The ATG39 mRNA levels in unstressed mig1 mig2 mutant. Wild-type (WT) and indicated mutant strains were grown at 25 °C until exponential phase. The ATG39 mRNA levels were quantified by qRT-PCR analysis, and relative mRNA levels were calculated using ACT1 mRNA. The values are plotted as the fold change from wild-type cells. The data show mean ± SEM (n = 4). **P < 0.01 as determined by Student’s t-test. (TIF) [file pgen.1009053.s009.tif]

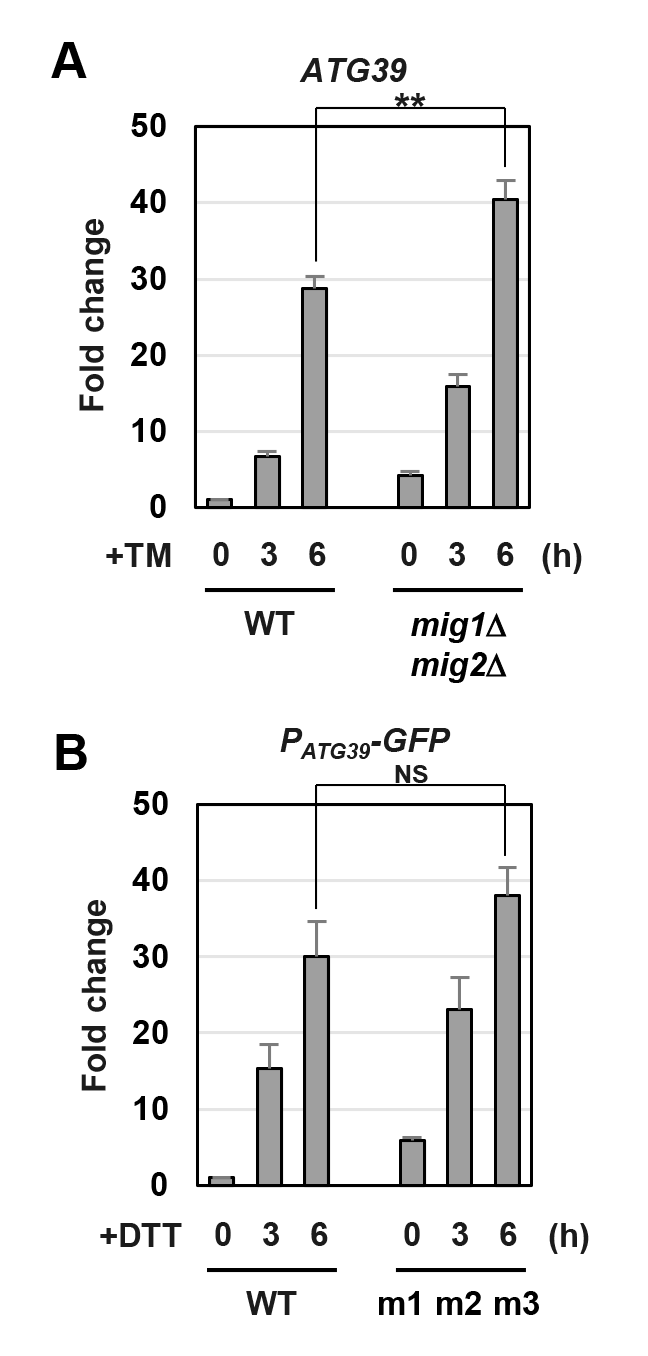

Supplement: S7 Fig — (A) The ATG39 mRNA levels in ER-stressed mig1 mig2 mutant. Wild-type (WT) and mig1 mig2 mutant strains were grown at 25 °C until exponential phase and treated with 3 μg/ml tunicamycin (TM) for the indicated time. The ATG39 mRNA levels were quantified by qRT-PCR analysis, and relative mRNA levels were calculated using ACT1 mRNA. The values are plotted as the fold change from wild-type cells at the time of TM addition. The data show mean ± SEM (n = 5). **P < 0.01 as determined by Student’s t-test. (B) Effects of mutations in putative Mig1/2-binding motifs on ER stress-induced ATG39 upregulation. Wild-type (WT) cells harboring the integration which expresses GFP under the control of wild-type or mutated ATG39 promoter were grown at 25 °C until exponential phase and treated with 6 mM dithiothreitol (DTT) for the indicated time. The GFP mRNA levels were quantified by qRT-PCR analysis, and relative mRNA levels were calculated using ACT1 mRNA. The values are plotted as the fold change from wild-type cells harboring the integration which expresses GFP under the control of wild-type ATG39 promoter at the time of DTT addition. The data show mean ± SEM (n = 4). NS, not statistically significant (P > 0.05), as determined by Student’s t-test. (TIF) [file pgen.1009053.s010.tif]

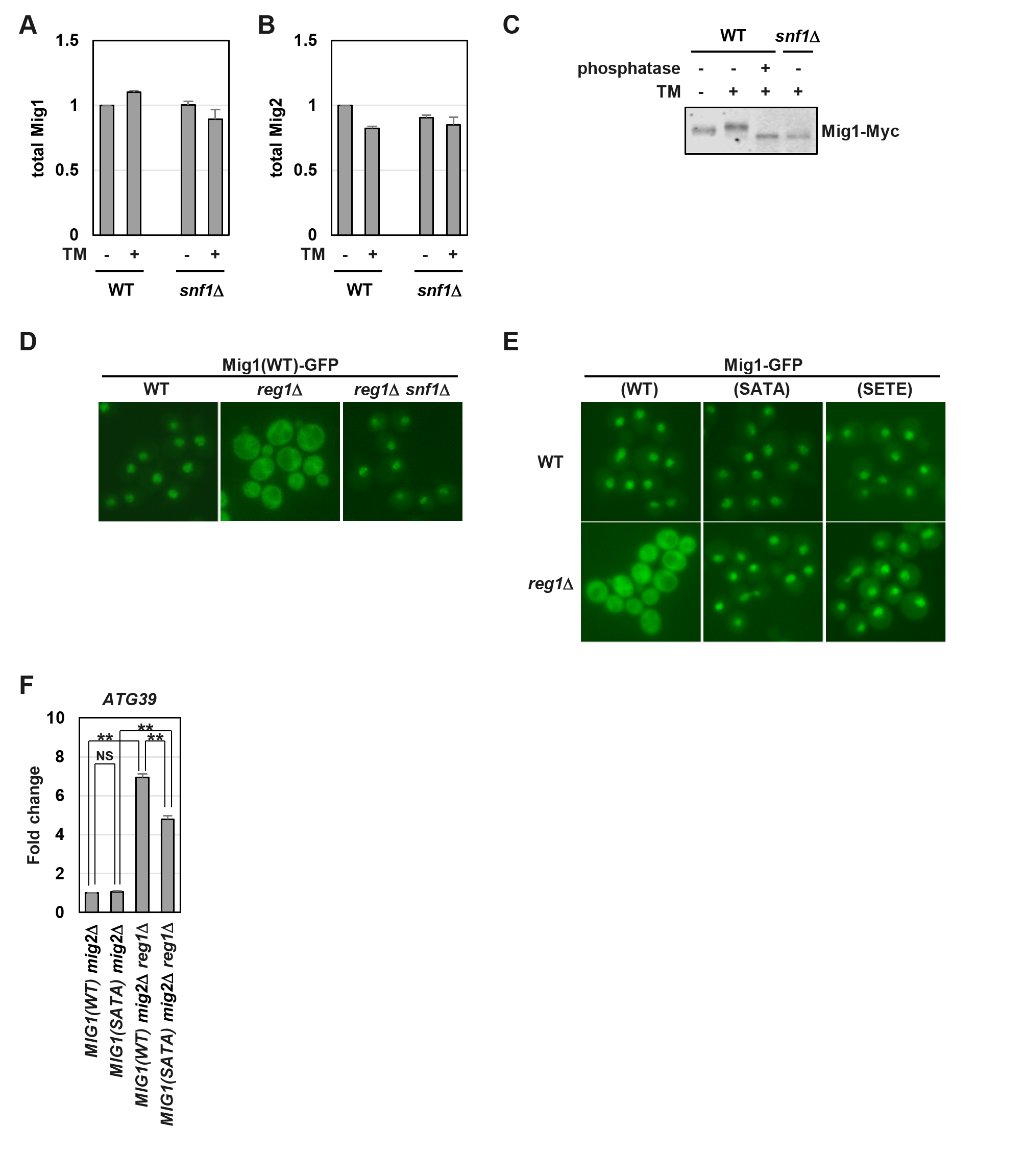

Supplement: S8 Fig — (A, B) The protein levels of Mig1 (A) and Mig2 (B). Wild-type (WT) and snf1 mutant strains harboring GFP-tagged MIG1 (A) or MIG2 (B) were grown at 25 °C until exponential phase, treated with 3 μg/ml tunicamycin (TM) for 3 hr. Extracts prepared from each cell were immunoblotted with anti-Myc antibodies. The intensities of Mig1-Myc and Mig2-Myc were measured and normalized to the Mcm2 level. The values are plotted as the fold change from wild-type cells at the time of TM addition. (C) Effects of the phosphatase treatment on Mig1. Wild-type (WT) and snf1 mutant strains harboring Myc-tagged MIG1 were grown at 25 °C until exponential phase and treated with 3 μg/ml tunicamycin (TM) for 3 hr. Extracts prepared from each cell were immunoblotted with anti-Myc antibodies. (D) Cellular localization of Mig1 in reg1 and reg1 snf1 mutants. Wild-type (WT) and indicated mutant strains harboring GFP-tagged MIG1 were grown at 25 °C until exponential phase and subjected to microscopy. (E) Cellular localization of Mig1 mutated in putative Snf1 phosphorylation sites. Wild-type (WT) and reg1 mutant strains harboring GFP-tagged MIG1 were grown at 25 °C until exponential phase and subjected to microscopy. (F) Effects of the phospho-defective mutation of Mig1 on ATG39 upregulation caused by reg1 mutation. The mig2 mutant strains harboring Myc-tagged wild-type or the phospho-defective mutant MIG1 were grown at 25 °C until exponential phase. The ATG39 mRNA levels were quantified by qRT-PCR analysis, and relative mRNA levels were calculated using ACT1 mRNA. The values are plotted as the fold change from MIG1(WT) mig2 cells. The data show mean ± SEM (n = 4). **P < 0.01 as determined by Student’s t-test. NS, not statistically significant (P > 0.05). (TIF) [file pgen.1009053.s011.tif]

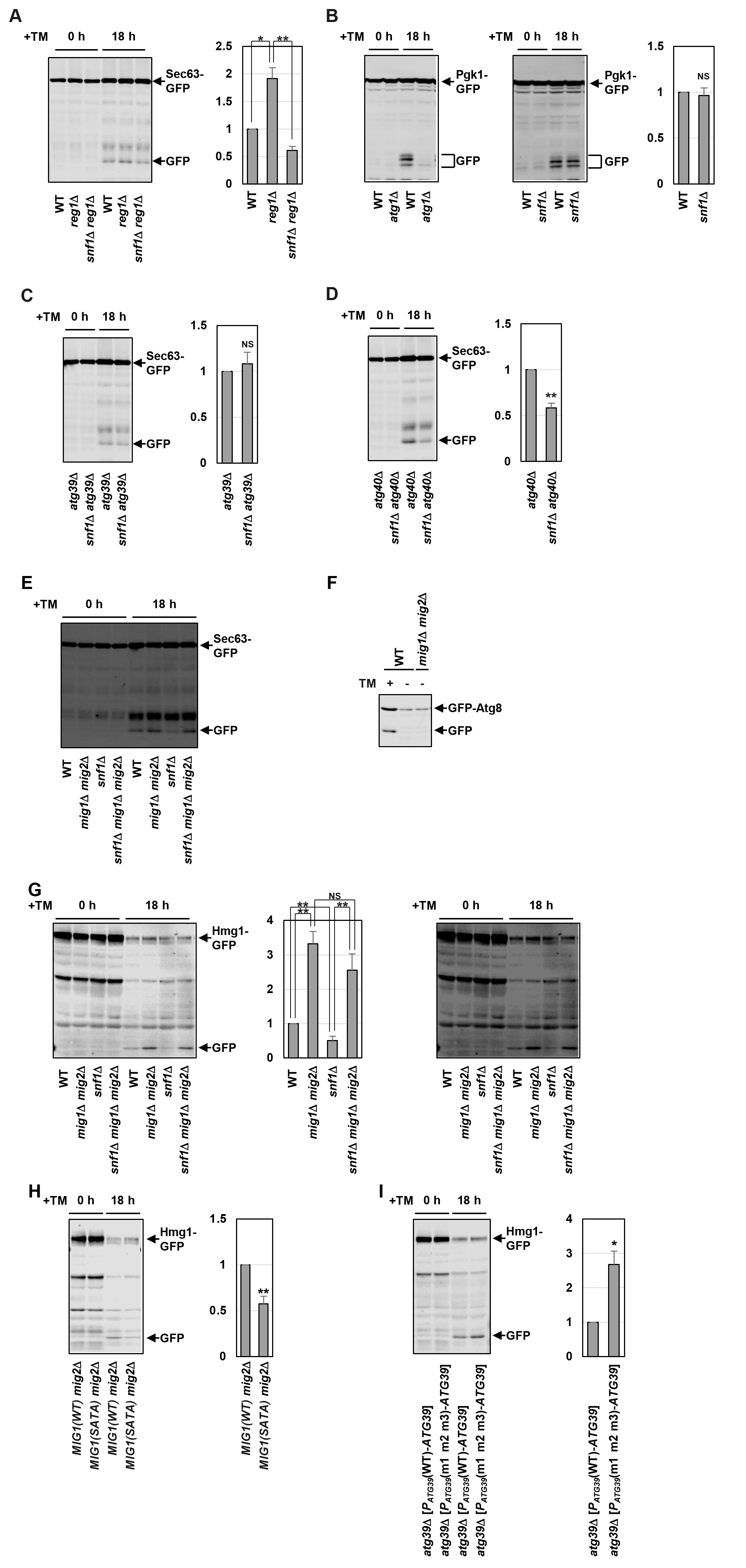

Supplement: S9 Fig — (A) Sec63-GFP degradation in Snf1-activated cells. Wild-type (WT) and indicated mutant strains harboring GFP-tagged SEC63 were grown at 25 °C until exponential phase and treated with 3 μg/ml tunicamycin (TM) for 18 hr. Extracts prepared from each cell were immunoblotted with anti-GFP antibodies. The intensities of free GFP were measured and normalized to the Sec63-GFP level. The values are plotted as the fold change from wild-type cells. The data show mean ± SEM (n = 3). *P < 0.05 and **P < 0.01 as determined by Student’s t-test. (B) Pgk1-GFP degradation in ER-stressed snf1 mutant. Wild-type (WT) and indicated mutant strains harboring GFP-tagged PGK1 were grown at 25 °C until exponential phase and treated with 3 μg/ml tunicamycin (TM) for 18 hr. Extracts prepared from each cell were immunoblotted with anti-GFP antibodies. The intensities of free GFP were measured and normalized to the Pgk1-GFP level. The data show mean ± SEM (n = 4). NS, not statistically significant (P > 0.05), as determined by Student’s t-test. (C, D) Effects of snf1 mutation on Sec63-GFP degradation in the atg39 and atg40 mutant cells. Indicated mutant strains harboring GFP-tagged SEC63 were grown at 25 °C until exponential phase and treated with 3 μg/ml tunicamycin (TM) for 18 hr. Extracts prepared from each cell were immunoblotted with anti-GFP antibodies. The intensities of free GFP were measured and normalized to the Sec63-GFP level. The values are plotted as the fold change from the atg39 mutant (C) or the atg40 mutant (D). The data show mean ± SEM (n = 4). **P < 0.01 as determined by Student’s t-test. NS, not statistically significant (P > 0.05). (E) Sec63-GFP degradation in mig1 mig2 mutant. A dark, high-contrast image of Fig 6C is shown. (F) GFP-Atg8 degradation in mig1 mig2 mutant. Wild-type (WT) and mig1 mig2 mutant strains harboring GFP-tagged ATG8 were grown at 25 °C until exponential phase and treated with or without 3 μg/ml tunicamycin (TM) for 6 hr. Extracts prepared from each cell [file pgen.1009053.s012.tif]

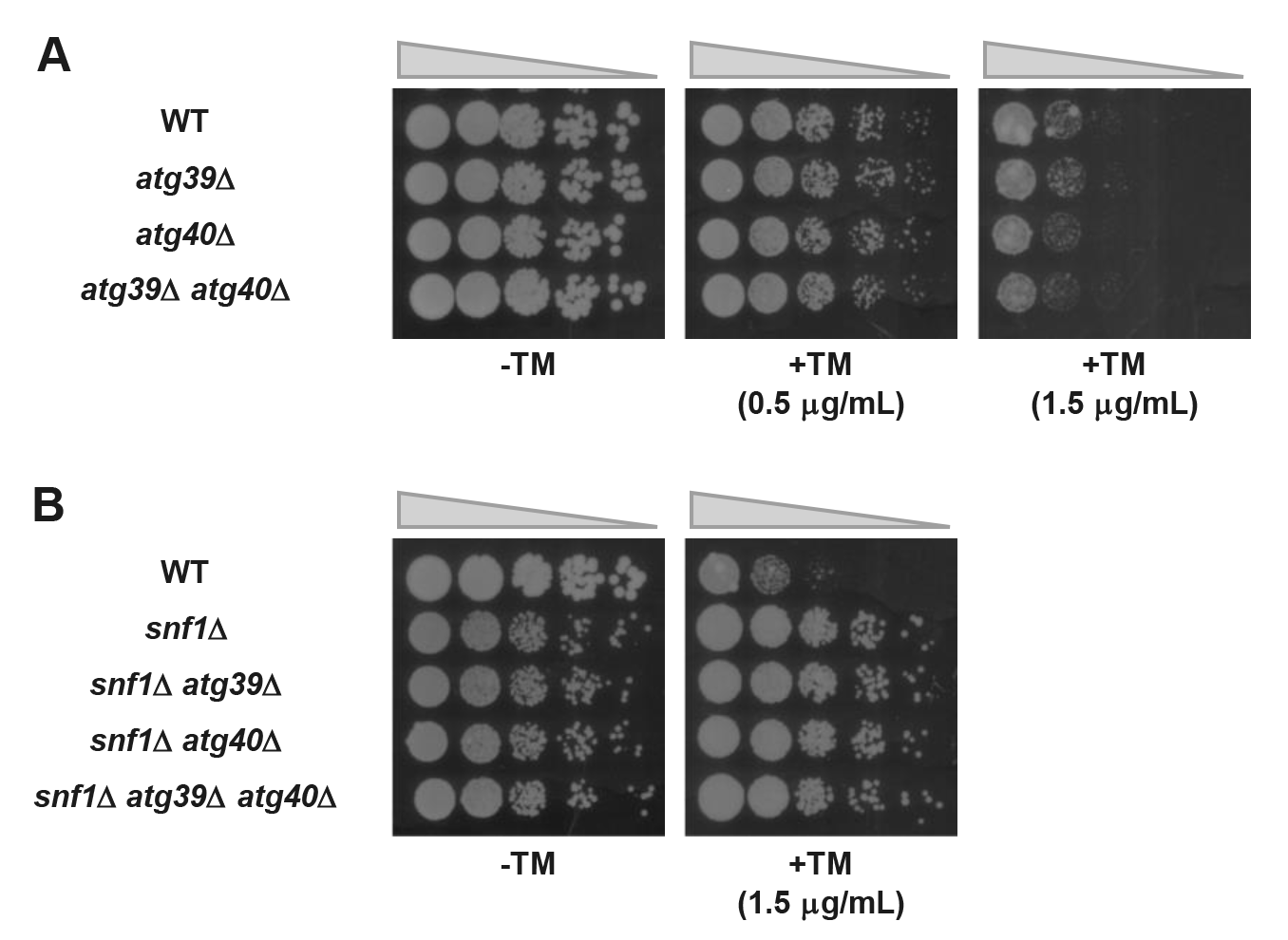

Supplement: S10 Fig — (A, B) Wild-type (WT) and indicated mutant strains were spotted onto YPD medium lacking or containing 0.5 or 1.5 μg/ml tunicamycin (TM) and incubated at 25 °C. (TIF) [file pgen.1009053.s013.tif]

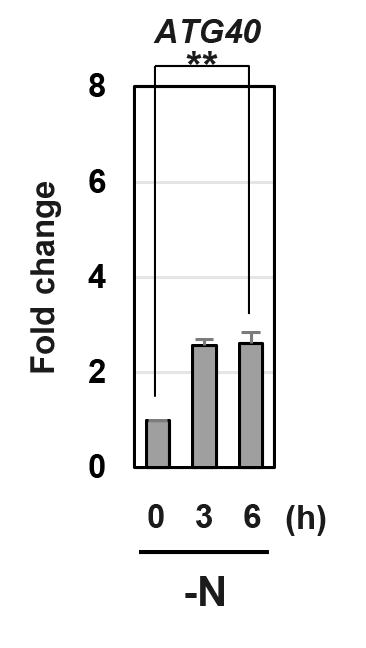

Supplement: S11 Fig — Wild-type strains were grown at 25 °C until exponential phase and then incubated under nitrogen starvation conditions for the indicated time. The mRNA levels were quantified by qRT-PCR analysis, and relative mRNA levels were calculated using ACT1 mRNA. The values are plotted as the fold change from untreated cells. The data show mean ± SEM (n = 3). **P < 0.01 as determined by Student’s t-test. (TIF) [file pgen.1009053.s014.tif]
