# Supplementary material for: Snf1 AMPK positively regulates ER-phagy via expression control of Atg39 autophagy receptor in yeast ER stress response
Source: PLoS Genet. 2020 Sep 28;16(9):e1009053. doi: 10.1371/journal.pgen.1009053 (PMC7544123; doi:10.1371/journal.pgen.1009053)
Supplement: Attachment — Submitted filename: Mizuno et al response 200719-1.doc [file pgen.1009053.s015.doc]

Dear Dr. David P. Toczyski,

Enclosed please find our revised manuscript entitled: " Snf1 AMPK positively regulates ER-phagy via expression control of Atg39 autophagy receptor in yeast ER stress response " (by Mizuno et al., PGENETICS-D-20-00376). Thank you for your e-mail of April 30, 2020 and the reviewers' comments on our manuscript. We appreciate these comments and they were helpful in improving the manuscript. We have conducted further experiments designed to address the comments of the reviewers, and we believe that we have now satisfactorily addressed almost all of the concerns they had raised. We hope that this revised manuscript is now acceptable for publication.

With best regards,

Tomoaki Mizuno

Paper: PGENETICS-D-20-00376

Authors: Mizuno et al.

Revised Title: Snf1 AMPK positively regulates ER-phagy via expression control of Atg39 autophagy receptor in yeast ER stress response

The manuscript has been revised in accordance with the comments raised by the editor and three referees. Responses to the comments are as follows:

**Our responses to the comments of the Editor**

**1.** *Rtn1, or some other resident ER protein, should be used in addition.*

As suggested, we examined degradation of GFP-tagged Rtn1, Yop1, Hmg1 and Src1. We show new results in revised Figures S2 and S9G-I, and mention them in the Results and Discussion sections (p. 8, line 152- p. 9, line 196, p. 17, line 413- p. 19, line 461, and p. 23, line 524- p. 24, line 576).

**2.** *The epistasis of Mig1/2 phosphomutants with snf1∆ would better explore your model.*

As suggested, we tried to generate a phospho-mimetic of Mig1, Mig1(SETE) by substitution of phosphorylatable serine/threonine residues with glutamates. However, Mig1(SETE) displayed the localization pattern similar to a phospho-defective form of Mig1, Mig1(SATA). This indicates that Mig1(SETE) acts as a phospho-defective mutant. Instead, we examined the phenotypes of Mig1(SATA) and found that they were similar to, but relatively mild, those observed in the *snf1* mutant. We show data from Mig1(SATA) in revised Figures 5E-F, 6D, S8E-F and S9H, and mention them in the results section (p. 16, line 370- p. 17, line 409 and p. 19, line 446- line 453). We believe that data from Mig1(SATA) strongly support our conclusion.

**3.** *Determining whether deletion of SNF1 also affects upregulation of ATG32/36 will allow the reader to understand better whether SNF1 functions uniquely in ER-phagy.*

As suggested, we examined the mRNA levels of *ATG32* and *ATG36* in the *snf1* mutant. Expression of *ATG32* and *ATG36* was unaffected by *snf1* mutation. We show new results in revised Figures S4C-D and mention them in the results and discussion sections (p. 11, line 249- p. 12, line 253).

**4.** *The missing reference noted by the first reviewer and other aspects of the text need to be amended.*

As suggested, we have cited references correctly and rewritten the text.

**Our responses to the comments of Reviewer #1**

**1.** *It has already been reported that Atg40 responds to ER stress (Cui et al. 2019 365, 53-60 Science). The Abstract, Introduction* *and Discussion needs to be rewritten to correct inaccurate statements regarding this point.*

As suggested, we have cited the reference correctly and rewritten the Abstract, Introduction and Discussion sections (p. 2, line 27- line 30, p. 5, line 102- line 105, and p. 24, line 584- line 588).

**2.** *The authors use one ER protein, Sec63, to do all their analysis. This is not the best marker to use for Atg39 as it is the nucleophagy receptor. Mochida et al. 2015 Nature showed that Atg39 primarily degrades the nucleus. Their analysis of Atg39 was done with proteins that mark the nuclear envelope (Hmg1), the inner nuclear membrane (Src1) and the nucleolus (Nop1). These markers do no label the cortical ER. A cortical ER marker, Rtn1, should also be analyzed as a negative control.*

As suggested, we tried to generate strains expressing the carboxyl-terminally GFP-tagged Hmg1, Src1, Nop1 or Rtn1. However, haploid cells harboring *Nop1-GFP* were inviable in our strain background. Therefore, we examined degradation of GFP-tagged Rtn1, Hmg1 and Src1. Of note, Rtn1-GFP degradation in response to ER stress was significantly reduced by *atg39* mutation. We show new results in revised Figures S2 and S9G-I, and mention them in the Results and Discussion sections (p. 8, line 152- p. 9, line 196, p. 17, line 413- p. 19, line 461, and p. 23, line 524- p. 24, line 576).

**3.** *The authors claim that Sec63-GFP degradation induced by tunicamycin, does not depend on Atg40. The data in Figure 1B, however, shows that Atg39 and Atg40 act synergistically to degrade Sec63.*

As suggested, we have rewritten this part (p. 9, line 174- line 178).

**4.** *The authors claim that Atg39 does not play a major role during nitrogen starvation is based on the finding that they do not see degradation of Sec63-GFP (Figure 1C). They need to look at other markers (see comment # 2) before drawing this conclusion.*

As suggested, we compared degradation of Rtn1-GFP, Hmg1-GFP and Src1-GFP with Sec63-GFP under nitrogen-starved conditions. We show new results in revised Figures S2L, M and N, and mention them in the Results and Discussion sections (p. 9, line 184- line 196 and p. 23, line 524- 539).

**5.** *I don’t understand why tunicamycin, a drug that blocks N-linked glycosylation, induces pexophagy, mitophagy and nucleophagy (Atg39).*

We discuss this point in the Discussion section (p. 22, line 503- 521).

**6.** *The authors state that rapamycin is “a compound that makes cells nitrogen-starved”. This is a* *misleading statement. Rapamycin is a drug that mimics starvation. Rapamycin and nitrogen starvation do not always stimulate the same autophagy pathways (i.e. the requirements for rapamycin induced and nitrogen starvation induced autophagy are not always the same).*

We apologize this misleading statement and have corrected it.

**7.** *The data on Snf1 and Mig1 and Mig2 are not always convincing.*

We first examined phosphorylation of Mig1 and Mig2 by their mobility pattern in SDS-PAGE. Their phosphorylation was upregulated by ER stress and inhibited by *snf1* mutation (revised Figures 5A-B, and S8C). We also examined the effect of the phospho-defective mutation in Mig1. The phospho-defective mutation enhanced Mig1 nuclear localization (revised Figure 5E) and reduced *ATG39* expression (revised Figures 5F and S8F) and degradation of Sec63-GFP and Hmg1-GFP (revised Figures 6D and S9H). We further discuss about seemingly inconsistent data in the Discussion section (p. 24, line 604- p. 25, line 637). Additionally, we show the modified model in revised Figure 7. We believe that additional data and modified description make our data convincing.

**Our responses to the comments of Reviewer #2**

**Major concerns:**

**1.** *A key part of their model is that Snf1 activation due to ER stress leads to the phosphorylation of Mig1/2 repressors and their nuclear export, thereby de-repressing Atg39 transcription. The supporting data for this idea, however, also reveal additional complexities that are not captured by their model. For example, in the triple (snf1 mig1 mig2) mutant (Fig 4C), ER stress still induces a handsome level of Atg39 transcriptional induction. The authors focus on the significance of the slight decrease in the maximum of this induction that is apparently Snf1-dependent (ie. relative to mig1 mig2) but to me it is more shocking how much inducibility persists that remains unaccounted for. Along the same lines, even though in mig1 mig2 there is a ~3-fold increase in basal Atg39 transcription (Fig 2B), there is also a ~20-fold Atg39 transcriptional induction due to ER stress. Given these complexities (ie. the apparent existence of undefined ER stress signaling routes to the Atg39 promoter (independent of Snf1), as well as from Snf1 to the Atg39 promoter (independent of Mig1/2), it would behoove the authors to further validate their model in the following two ways. First, define the effect of Snf1 phosphosite mutations on Mig1/2 on basal and ER-stress induced Atg39 mRNA levels. Second, use a minimal Atg39 promoter construct (e.g. crippled promoter with MBM region) to provide a transcriptional reporter that fully captures the key aspect of their model but excludes other undefined transcriptional regulation factors that complicate the picture they are describing.*

As suggested, we generated a phospho-defective mutant of Mig1, Mig1(SATA), by substitution of phosphorylatable serine/threonine residues with alanines and examined its phenotypes. We found that Mig1(SATA) displays the phenotypes similar to, but relatively mild, those observed in the *snf1* mutant. We show data from Mig1(SATA) in revised Figures 5E-F, 6D, S8E-F and S9H, and mention them in the results section (p. 16, line 370- p. 17, line 409 and p. 19, line 446- line 453). We also tried to generate a phospho-mimetic of Mig1, Mig1(SETE), but Mig1(SETE) seemed to be a phospho-defective mutant (revised Figure S8E).

As suggested, a minimal promoter construct is a powerful tool to investigate transcriptional regulation; for example, the UPRE reporters have accelerated our understanding of the unfolded protein response (UPR) mechanism. However, we feel it difficult to construct a minimal *ATG39* promoter construct by following reasons. First, Mig1/2 repress target genes cooperatively with the Cyc8-Tup1 corepressor complex. Mig1 is not required for tethering the Cyc8-Tup1 corepressor complex to the promoter (Papamichos-Chronakis *et al.* EMBO Rep 2004). The Cyc8-Tup1 corepressor complex does not bind to DNA directly (Komachi *et al.* Genes Dev 1994, Smith *et al.* Genes Dev 1995). These suggest that another factor and its binding site are required for Mig1-mediated repression. Second, the *SUC2* and *GAL1* genes are well-known as a Mig1 target gene since 1990s. However, their minimal promoter constructs are not utilized as far as we know. Third, we recently found the sequence that is located near Mig1/2-binding motif and functions in *ATG39* upregulation. Therefore, we consider it difficult to develop a minimal *ATG39* reporter.

As pointed out by the reviewer, our data suggest not only the Snf1-Mig1/2-*ATG39* pathway but also the Mig1/2-independent and Snf1-independent pathways. In the Discussion section of revised manuscript, we carefully explain these mechanisms with citing the references (p. 26, line 604- p. 27, line 637). Furthermore, we show the modified model in revised Figure 7. We believe that additional data and modified description in revised manuscript significantly improve the understanding of our research.

**2.** *Figure 5 provides evidence for* *a subcellular localization change of Mig1 and Mig2 under ER stress (TM treatment). It is not clear to me that this relatively small 2-fold change is sufficient to drive the associated Atg39 transcriptional change (presumably close to an order of magnitude fold induction by comparison to similar conditions in other experiments shown) they are reporting. The authors should consider examining Mig1/2 protein level and phosphorylation status to rule out other contributions to Mig1/2-regulated Atg39 expression beyond nuclear export.*

We examined the protein levels and phosphorylation of Mig1 and Mig2, show new results in revised Figures 5A-B and S8A-C, and mention them in the Results section (p. 15, line 344- line 358). Seeming inconsistency between a subcellular localization change of Mig1/Mig2 and the associated Atg39 transcriptional change pointed out by the reviewer is perhaps derived from inhibition of the physical interaction between Mig1/2 and the repressor complex that is mediated by Snf1 phosphorylation, as shown previously (Papamichos-Chronakis *et al.* EMBO Rep 2004). This inhibition occurs in the nucleus and does not require nuclear export of Mig1. We have cited this paper and discuss our data in the Discussion section (p. 26, line 604- line 617). Additionally, we show the modified model in revised Figure 7.

**Minor concerns:**

**1.** *The authors acknowledge that “It is possible that the long duration of ER stress is required for Atg39 to express enough to induce ER-phagy.” but they should show the kinetics of the key steps in their model (1. Snf1 kinase activation; Mig1/2 nuclear export; Atg39 transcriptional induction measured as mRNA level; Atg39 protein induction) by showing them together in relationship to one another. As it stands, it is hard to define the nature of the “slow step” in their model. For example, in Fig 2, the mRNA level of Atg39 already increased by ~50-fold after at 6 hrs tunicamycin treatment, but no free GFP band was detected in the Sec63-GFP processing assay (Fig 1).*

We consider that our data are not enough to make our previous model plausible and then have rewritten this part (p. 20, line 474- line 487).

**2.** *The authors make the broad claim that Atg39 makes the dominant contribution to ER stress-induced ER-phagy. This is in contrast to the dominant role of Atg40 in ER-phagy under nitrogen starvation. Given that previous work has shown that Atg40 is enriched in highly curved ER membranes (tubules and sheet edges vs sheets) whereas Sec63 is preferentially found in ER sheets, it is conceivable that the Sec63-GFP processing assay could be a biased metric of ER-phagy. The authors could consider defining the autophagic flux of the two ER-phagy receptors themselves as a potentially more realistic representation of their contribution to ER-phagy induced by ER stress.*

As suggested, we examined degradation of Atg39-GFP and Atg40-GFP in response to ER stress and nitrogen starvation. Free GFP produced from Atg39-GFP and Atg40-GFP was clearly detected in cells under nitrogen-starved conditions as shown in supplementary data A. Of note, Atg39-GFP produced many free GFP compared with Atg40-GFP. This result is unexpected for us, because we considered that Atg40 is more important for nitrogen starvation-induced ER-phagy than Atg39 based on the data showing degradation of Sec63-GFP and Rtn1-GFP (revised Figures 1C and S2L). Perhaps, the activity per molecule to degrade cargoes may differ between autophagy receptors. Furthermore, we hardly detected intact Atg39-GFP and free GFP produced from Atg39-GFP and Atg40-GFP in response to ER stress. Difficulty of detecting Atg39-GFP and free GFP in ER stress response may be partly due to lower *ATG39* induction (compare revised Figure 2A with revised Figure 3E) and activity of the core autophagy machinery (supplementary data B and C). To date, we cannot explain these observations sufficiently. We discuss the mechanisms of yeast ER-phagy in the Discussion section (p. 23, line 540- p. 24, line 576).

**3.** *There is visible new, lower mobility Atg39-myc species in Fig 3B that appears associated with ER stress. The authors should at the very least comment in the figure legend whether this species corresponds to a form of Atg39 (vs. a cross-reacting anti-Myc species also present in Atg39 control lacking Myc tag).*

As suggested, we show the western blot data including samples extracted from cells expressing non-tagged Atg39 (revised Figure S5A). This data shows that the new, lower mobility Atg39-Myc species pointed out by the reviewer corresponds to a form of Atg39. The lower mobility Atg39-Myc species was detected even in the extract treated with alkali phosphatase (revised Figure S5B). We mention these results in the Results and Discussion sections (p. 11, line 239- line 243 and p. 23, line 559- line 576).

**4.** *It would be helpful for completion to include the changes of Atg40 mRNA level in snf1∆ cells under TM treatment in supplementary figure, if these data have been already collected.*

As suggested, we show *ATG40* mRNA levels in the *snf1* mutant treated with tunicamycin in revised Figure S4B.

**5.** *Actual experimental evidence of direct binding of Mig1/2 to the Atg39 promoter would help provide further support for their model. Could the authors comment on whether this is the case based on mining of published large-scale ChIP-seq data sets.*

Large-scale ChIP-chip experiments have identified the genome-wide binding sites of a large number of yeast transcription factors (Lee *et al.* Science 2002, Harbison *et al.* Nature 2004). In these studies, the significant interaction between Mig1/2 and *ATG39* promoter was not unfortunately detected. However, the possibility that Mig1/2 bind to the *ATG39* promoter could not be excluded, because the binding of Mig1 to its well-known target genes such as *SUC2* and *GAL1* had frequently missed in these studies. We would like to investigate direct binding of Mig1/2 to the *ATG39* promoter in the future to reveal the detailed mechanism underlying transcriptional regulation of *ATG39.*

**6.** *Please comment on the visible band shift to higher molecular weight of Sec63-GFP after 18 hr TM treatment (e.g. Fig 6D, 6E).*

We recently noticed that the western blot data is clearly improved using WIDE RANGE Gel Preparation Buffer(4x) for PAGE (Nakalai) (p. 31, line 736- line 737). Therefore, we have re-obtained almost all western blot data about degradation of GFP-tagged protein using this reagent. To obtain revised Figures 6C and E, we reused samples that was used in original Figures 6D and E but could not observe the band shift of Sec63-GFP. It may be possible that ER stress causes post-translational modifications of Sec63-GFP, which can be detected only under specific experimental conditions.

**7.** Others.

As suggested, we have rewritten the descriptions pointed out by the reviewer.

**Our responses to the comments of Reviewer #3**

**Major concerns:**

**1.** *The major readout for ER-phagy throughout the paper is Sec63-GFP cleavage. This runs into the risk of potentially studying only the effect of Snf1/Mig1/2 regulation on Sec63 degradation rather than overall ER degradation. The authors should show the degradation of other ER resident proteins. This does not need to be done for all ER-phagy assays but the authors should at least be able to demonstrate that the degradation of another ER protein follows the same trend as Sec63 under ER stress vs nitrogen starvation with Atg39/40 mutations (similar experimental set up as Fig 1B and C).*

As suggested, we examined degradation of GFP-tagged Rtn1, Yop1, Hmg1 and Src1. We show new results in revised Figures S2 and S9G-I, and mention them in the Results and Discussion sections (p. 8, line 152- p. 9, line 196, p. 17, line 413- p. 19, line 461, and p. 23, line 524- p. 24, line 576).

**2.** *In Figure 6, the authors used a PGK-GFP cleavage assay to show that Snf1 KO does not affect bulk autophagy. However, the authors showed that ER stress also induces mitophagy and pexophagy. It seems important to investigate whether KO of Snf1 also affects upregulation of Atg32 and Atg36. This will shed light on whether Snf1 is a specific ER-phagy inducer or a global organelle-autophagy inducer during ER stress.*

As suggested, we examined the mRNA levels of *ATG32* and *ATG36* in the *snf1* mutant. Expression of *ATG32* and *ATG36* was unaffected by *snf1* mutation. We show new results in revised Figures S4C-D and mention them in the Results sections (p. 11, line 249- p. 12, line 253).

**3.** *In Fig 5A, the fluorescence intensity ratio (N/C) data for Mig1 is very subtle, especially if compared to control versus TM treatment of both WT and Snf1KO. Is it possible that Snf1 regulates Mig1 in a different fashion than Mig2 (i.e. apart from nuclear export)?*

We have cited the references and discuss this point in the Discussion section (p. 26, line 604- line 617).

**4.** *The authors draw a great deal of conclusions from previous literature showing that phosphorylation of Mig1&2 causes nuclear export. But the link between Mig1/2 phosphorylation and ER-phagy is not formally tested in this paper. This hypothesis should be checked, since it’s a major part of their model. One suggestion would be to generate phosphomimetic Mig1 and Mig2 mutants in Snf1 KO cells to see if Mig1&2 are driven out of the nucleus and trigger ER-phagy even in the absence of Snf1 KO.*

As suggested, we tried to generate a phospho-mimetic of Mig1, Mig1(SETE) by substitution of phosphorylatable serine/threonine residues with glutamates. However, Mig1(SETE) displayed the localization pattern similar to that of a phospho-defective form of Mig1, Mig1(SATA). This indicates that Mig1(SETE) acts as a phospho-defective mutant. Instead, we examined the phenotypes of Mig1(SATA) and found that they were similar to, but relatively mild, those observed in the *snf1* mutant. We show data from Mig1(SATA) in revised Figures 5E-F, 6D, S8E-F and S9H, and mention them in the results section (p. 16, line 370- p. 17, line 409 and p. 19, line 446- line 453). We believe that data from Mig1(SATA) strongly support our conclusion.

**5.** *The authors’ data very nicely shows the strong effect of Snf1 and Mig1/2 on transcription of Atg39. However, Snf1 and Mig1/2 have only a minor effect on ER-phagy. This is apparent in Fig 6A/D where Snf1 KO showed a statistically significant but relatively small inhibition of ER-phagy. So Snf1 and Mig1/2 regulate the ER-phagy receptors, but why does this not translate into marked effects on ER-phagy? The authors should try to delve deeper into how the transcriptional regulation of Atg39 correlates with ER-phagy.*

As shown in original Figure 1B, *atg39* single mutation only halved Sec63-GFP degradation in response to ER stress, due to the significant contribution of Atg40. This Atg40 contribution makes the effects of *snf1* mutation and *mig1 mig2* double mutations on Sec63-GFP degradation relatively small (revised Figures 6A and C). In the revised manuscript, we found the stronger effect of *atg39* mutation on degradation of Hmg1-GFP compared with Sec63-GFP (revised Figures 1B and S2J). Consistently, we could find that Hmg1-GFP degradation is significantly downregulated and upregulated by *snf1* mutation and *mig1 mig2* double mutations, respectively. We show these new results in revised Figures S9G-I and mention them in the Results section (p. 17, line 413- p. 19, line 461).

**6.** *In Fig 4C, the data do not quite fit with the model they proposed. If the sole function of Snf1 during ER-phagy is to remove Mig1 and Mig2 from the Atg39 promoter, triple KO of Snf1/Mig1/Mig2 should phenocopy Mig1/Mig2 double KO. The fact that the triple mutant showed less ATG39 activation compared to WT or Mig1+2 mutant suggests that Snf1 also upregulates ATG39 via an additional mechanism beyond Mig1 and Mig2. The authors did point this out this during discussion but I think this should also be noted in the result section and further expanded in the discussion on what other potential mechanism that could be. And this additional function of Snf1 should also be reflected in the schematic of Fig 7.*

As suggested, we have rewritten this part in the Results section (p. 14, line 307- line 318) and discuss the relationship between Snf1 and Mig1/2 in the Discussion section (p. 14, line 309- line 320). Furthermore, we show the modified model in revised Figure 7.

**Minor concerns:**

**1.** *The authors showed in Fig.2 that ER stress (TM and DTT) causes transcriptional up regulation of all ATG receptors including ER, mitochondria and peroxisomes. Does this mean that ER stress acts as a general autophagy inducer? While this is not the main focus of the paper, this is an unexpected finding and the authors should address this observation in the discussion.*

As suggested, we discuss this point in the Discussion section (p. 21, line 503- 521).

**2.** *There is a distinct difference in the abundance of full length Sec63-GFP band between TM versus nitrogen starvation (compare Fig 1B and C). The authors should address what this means in the discussion.*

We recently noticed that the western blot data is clearly improved using WIDE RANGE Gel Preparation Buffer(4x) for PAGE (Nakalai) (p. 31, line 736- line 737). Therefore, we have re-obtained almost all western blot data about degradation of GFP-tagged protein using this reagent. To obtain revised Figures 1B-C, we reused samples that was used in original Figures 1B-C. However, a distinct difference in the abundance of full length Sec63-GFP band between TM versus nitrogen starvation was not observed. One possibility to explain these data is that nitrogen starvation may cause post-translational modifications of Sec63-GFP and these modifications may inhibit transfer of Sec63-GFP from polyacrylamide gels to PVDF membranes in our previous procedure.

**3.** *Since double KO of Mig1 and Mig2 resulted in upregulation of Atg39 even at basal state (e.g. Fig 4A), one might expect that ER-phagy should also be enhanced at basal state but the authors did not observe this in Fig 6D. The authors should explain/discuss this.*

We examined GFP-Atg8 degradation in the *mig1 mig2* double mutants and found that *mig1 mig2* double mutation does not cause aberrant activation of the core autophagy machinery under unstressed conditions. Therefore, *ATG39* upregulation caused by *mig1 mig2* double mutation fail to induce ER-phagy under unstressed conditions. We show this result in revised Figure S9F, and mention it in the Results section (p. 19, line 440- line 446). Additionally, we show the modified model in revised Figure 7.

**4.** *In line 221-222 of the main text - The sentence ‘…rapamycin, a compound that makes cells nitrogen-starved’ is misleading since rapamycin does not cause nitrogen starvation but instead it mimics the downstream signal activation during nitrogen starvation.*

We apologize this misleading statement and have corrected it.

**5.** *In general, I feel the authors need to explain their findings in more detail in the discussion. I also felt this paper delves deep into ER-phagy in yeast, but there is almost no connection made to the burst of recent papers on mammalian ER-phagy.*

As suggested, we have rewritten the Discussion section entirely to explain our findings in more detail and how our findings contribute to the understanding of ER-phagy.
